# Surgical Excision of Carotid Body Tumor at an Early Stage Has Best Outcome: Result of 22 Cases along with Literature Review

**DOI:** 10.3400/avd.oa.20-00084

**Published:** 2020-12-25

**Authors:** Rashid Usman, Muhammad Jamil, Aaiza Aman

**Affiliations:** 1 Department of Vascular Surgery, Combined Military Hospital, Lahore Cantt, Pakistan; 2 Department of Vascular Surgery, Combined Military Hospital, Rawalpindi Cantt, Pakistan; 3 Department of Surgery, Fauji Foundation Hospital, Rawalpindi, Pakistan

**Keywords:** carotid body tumor, chemodectoma, Shamblin, carotid surgery

## Abstract

**Objective**: The objective of this study is to share our experience of early surgical excision of highly vascular carotid body tumor (CBT) and to correlate it with current literature.

**Materials and Methods**: Data of all consecutive patients diagnosed with CBT from September 2011 to September 2018, who underwent surgical excision, was analyzed.

**Results**: Of the 22 cases with mean age of 42±standard deviation (SD) 6 years and female to male ratio of 1.2 : 1, 68.1% (n=15) of the tumors were on the right side. There were 13.6% (n=3) Shamblin I, 77.2% (n=17) Shamblin II, and 9% (n=2) Shamblin III tumors. Complete excision without vascular reconstruction was achieved in 63.6% (n=14), while patch plasty with Dacron graft was noted in 29.4% (n=5) and interposition Dacron grafting 13.6% (n=3). Peroperative vascular shunt was deployed in 13.6% (n=3) of cases. Transient neuropraxia of the hypoglossal nerve was noted in 13.6% (n=3) of cases, while permanent drooping of the lower lip was noted in 4.5% (n=1). There was no ischemic stroke. The mortality rate was zero, and no recurrence was recorded in mean follow-up of 24±SD 3 months.

**Conclusion**: Complete surgical excision of CBT at an early stage, regardless of size, is associated with the best outcome.

## Introduction

Carotid body tumors (CBTs) are rare, slow-growing, hypervascular neuroendocrine (paragangliomas or chemodectomas) benign tumors with tendency for malignant transformation, arising from the carotid body near the carotid bifurcation, with an incidence of 0.002%.^1–3)^ The incidence is higher in high-altitude areas due to long-term hypoxia which results in hyperplasia of the carotid body.^3)^ Most of the time, the tumor is noticed as a slow-growing lump in the upper neck or incidentally discovered in some radiological investigation.^2)^ The patient may present with neurological symptoms such as cranial nerve palsies and carotid sinus syndrome resulting in dizziness and syncope.^4)^ Digital subtraction angiography is the gold standard for the diagnosis of CBTs.^3)^ The curative treatment of choice is complete surgical resection.^5)^ Data on the management of CBT in South Asian population is limited. The purpose of our study is to share our experience regarding presentation, diagnosis, treatment, and outcome of CBTs in our population along with review of literature.

## Materials and Methods

After approval from the ethical committee/institutional review board [Ser No. 76/05/20 (22)], the data of all patients operated for CBT from September 2011 to September 2018 was analyzed. All these patients were operated by the same surgical team across various level 1 military hospitals of Pakistan, namely, Combined Military Hospital Lahore, Peshawar, Quetta, and Rawalpindi. Patients who were found unfit for surgery, who denied surgery, with incomplete case notes, and who lost to follow-up were excluded.

Patient demographics, clinical presentation, investigations, operative findings, operative procedure, complications, and follow-up were recorded from the case notes. All patients underwent a carotid duplex scan as a preliminary investigation followed by computerized tomographic angiography (CTA) of the carotids. Based on CTA findings, they were categorized into Class I, II, or III according to the Shamblin classification system.^6,7)^

The data was analyzed using Statistical Package for Social Sciences Version 20.0. Numerical data, such as age, was presented as mean and standard deviation (SD), and categorical data, such as gender, was recorded as frequency and percentage.

## Results

**Figure 1:**
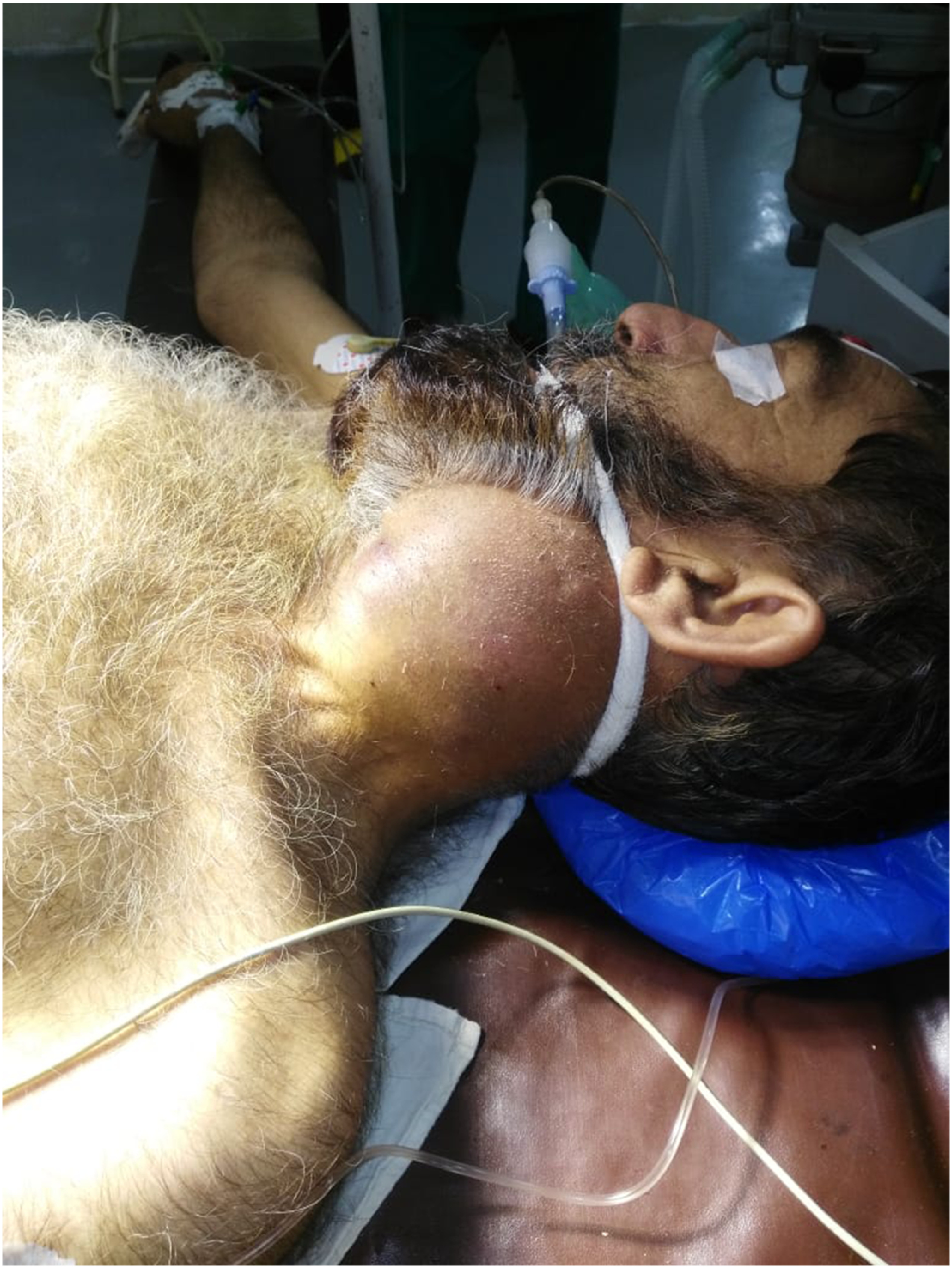
Fig. 1 Preoperative picture showing neck lump due to carotid body tumor (Shamblin class III).

Two patients with poor cardiac reserves were unfit for anesthesia, while one patient denied surgery. A total of 22 cases fulfilling the inclusion criteria were included in this study. Mean age at the time of presentation was 42±SD 6 (range, 35–56) years, and there were 12 women, with a female to male ratio of 1.2 : 1. In total, 68.1% (n=15) of patients had CBT on the right side, and 31.8% (n=7) had CBT on the left side. The main presenting symptom was painless slow-growing lump on the side of the neck (**Fig. 1**). Mean time from noticing a painless lump to first presentation was 5±SD 1 year in 91% (n=20) of patients, while it was 3±SD 3 months in patients who had dizziness/syncope along with a lump (9%, n=2) (**Table 1**).

**Table 1:** Table 1 Demographics and clinical presentation of patients with carotid body tumor

Age	42±SD 6 (range, 35–56) years
Gender	Male (n=10) Female (n=12)
Side affected	Right (n=15) Left (n=7) Bilateral (n=0)
Clinical presentation	Painless lump only (n=20)
	Painless lump with dizziness/syncope (n=2)
	Painless lump with cranial nerve palsy (n=0)
Patients from high-altitude (>7000 feet from sea level)	95.4% (n=21)

SD: standard deviation

There were 13.6% (n=3) Shamblin I, 77.2% (n=17) Shamblin II, and 9% (n=2) Shamblin III tumors. All patients were offered surgical excision. All the risks associated with surgery and the potential use of peroperative vascular shunt and graft (patch or interposition segment) were explained to patients. Informed consent of surgery was obtained including the permission to extend incision and maneuvers to expose the carotids toward the base of the skull for distal control if needed. Mean duration from diagnosis to operation was 10±SD 2 days.

All patients received general anesthesia. The tumor was approached through a standard vertical incision along the anterior border of the sternocleidomastoid muscle extending from the angle of the mandible to the sternoclavicular joint. After proximal and distal controls, bipolar diathermy was used to control the bleeding from the small feeders, while sub-adventitial dissection plane was created between the tumor and carotids.

**Figure 2:**
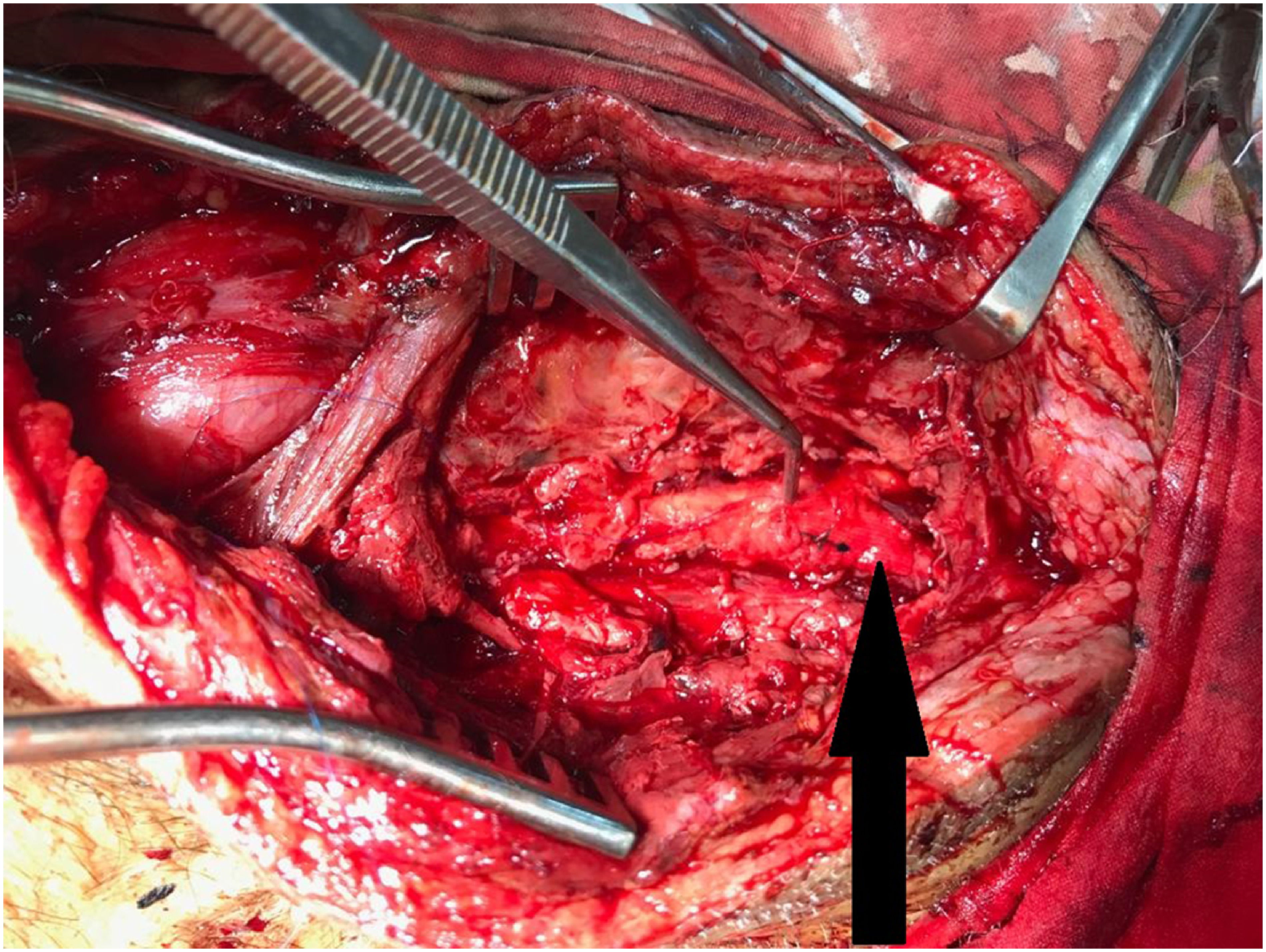
Fig. 2 Dacron patch plasty of the carotids after excising Shamblin class II carotid body tumor.

Complete excision was achieved without any inadvertent injury to the carotids in 63.6% (n=14) of patients, including all Shamblin I and 64.7% (n=11) Shamblin II patients. Of the rest of Shamblin II class, 29.4% (n=5) needed patch plasty with Dacron graft (**Fig. 2**), and 5.8% (n=1) needed an interposition graft between the common carotid artery (CCA) and internal carotid artery (ICA). Complete excision in the sub-adventitial plane was not possible in both patients with Shamblin III tumors. We excised the tumor along with the involved CCA, ICA, and external carotid artery (ECA), and reconstruction was done using an end-to-end Dacron interposition graft between ICA and CCA while ECA was ligated in both cases. In all three patients that need interposition grafting, Javid vascular shunt was deployed to restore the circulation to ICA during reconstruction. Mean shunt time was 50±SD 10 min. Mean operation time was 125±SD 20 (range, 100–180) min.

Transient motor neuropraxia of the hypoglossal nerve resolving within 3 months was noted in 13.6% (n=3) of cases. These include both Shamblin III tumors and one Shamblin II tumor. It is worth noting that all these three cases needed Dacron interposition grafting. One patient (4.5%) had permanent drooping of the lower lip due to injury to the marginal mandibular branch of the facial nerve. This patient had huge Shamblin III tumor that needs extension of the incision cranially. Our data suggests that operative difficulty and peroperative injuries were higher in Shamblin III tumor or huge tumors that need vascular reconstructions. It is worth noting that none of our patient developed ischemic stroke. The postoperative recovery was mostly uneventful. In 9% (n=2) there was small self-limiting non-expanding hematoma which resolved spontaneously. One patient had hematoma which was aspirated under ultrasound guidance. There was zero mortality. After analyzing all excised specimen, the diagnosis of CBT with no malignant transformation was confirmed. Minimum follow-up period was 18 months with a mean follow-up of 24±SD 3 months. No recurrence was recorded during this follow-up period.

## Discussion

Carotid body initially described by von Haller in 1743 is a chemosensitive organ located in the adventitia of carotid bifurcation, which plays an important role in controlling blood pressure and body temperature.^5,8,9)^ CBTs are rare, slow-growing, hypervascular neuroendocrine benign tumors which arise from the carotid body with an overall incidence of 0.002%.^1–3)^ However, it is still the most common type of paraganglioma and the only disease affecting the carotid body.^1)^

The incidence of CBTs is higher in high-altitude areas and is thought to be related to hyperplasia of the carotid body due to long-term hypoxia.^8–10)^ In our study, 95.4% (n=21) of patients were from high-altitude areas (>7000 feet from sea level). CBTs are more common in women, with male to female ratio of 1 : 1.9,^10)^ and our study also showed similar preponderance with male to female ratio of 1 : 1.2.

Mostly the tumor presents as a slow-growing painless mass in the upper part of the anterior triangle of the neck which may sometimes be associated with pain and dysphagia.^2)^ Some patients (>10%) may present with carotid sinus syndrome characterized by bradycardia, hypotension, and syncope.^2–4)^ All of our patients noticed a painless lump in the anterior part of the neck just below the angle of mandible. Only two patients (9%) presented with lump along with dizziness/syncope. The tumor was clinically of larger size in them (>5 cm). Furthermore, cranial nerve palsies of the vagus, hypoglossal, trigeminal, and facial nerves in descending order of frequency may be present in less than 10% of the tumors^6)^; however, none of our patients had cranial nerve palsy on presentation.

In Pakistan, due to its high prevalence, tuberculosis is one of the most common causes of neck mass, and it is often referred to a surgeon for an excision biopsy for the purpose of getting a histological diagnosis. It is of utmost importance for the surgeon to rule out rare causes of neck mass like CBT. If a CBT is clinically suspected, a duplex scan should be done to rule out the diagnosis.^11)^ Blind biopsy may cause excessive bleeding, nerve damage, dissemination, pseudoaneurysm, and even carotid thrombosis.^12,13)^ Digital subtraction angiography is considered as the gold standard for the diagnosis of CBTs.^3)^ CT/MRI is done to see the size and invasiveness of tumor.^10)^ In all our patients, CTA of the carotid arteries was done for definitive diagnosis and grading.

In 1971, Shamblin classified CBTs into three categories: type I is small tumor partially encasing carotids and can easily be peeled off, type II encases completely but can still be easily peeled off, while type III encases the carotids, hence requiring vascular resection.^7,8)^ However, this classification did not describe how deeply the tumor eroded the wall of the carotids. Hence, Luna-Ortiz et al. suggested a modification and labeled all those tumors which are clinically or histologically infiltrating the adventitia of the vessel regardless of tumor size as Shamblin IIIb.^14)^ In our study, 77.2% (n=17) of patients presented with Shamblin type II, while 9% (n=2) presented with Shamblin type III. Of these, there was only one confirmed Shamblin IIIb on histopathology.

Complete surgical excision is still the choice of curative treatment of CBTs. After the first surgery of CBT by Albert in 1889, Gordon-Taylor in 1940 introduced sub-adventitial dissection with ligation of small feeders to devascularize the tumor bed.^15,16)^ The craniocaudal dissection technique was introduced in 2008 by van der Bogt et al. to reduce blood loss and perioperative morbidity.^17)^ To start with, we also performed sub-adventitial dissection in craniocaudal fashion to devascularize and dissect the tumor from its bed. However, it was impossible to create a safe sub-adventitial dissection plane in three cases; hence, a decision was taken to excise the carotids along with the tumor and perform vascular reconstruction. The rate of vascular injury is higher in Shamblin III tumors. We had vascular injury in 22.7% (n=5) Shamblin II tumors, which was managed by Dacron patch plasty. Although controversial, preoperative embolization is beneficial in large-sized tumors to decrease the vascularity.^18,19)^ We did not perform preoperative embolization in any of our patients due to lack of such facilities in our centers.

Perioperative cranial nerves injury and stroke during resection of CBTs is 0%–7% and 1%, respectively. A meticulous surgical technique, heparin, intraluminal shunt, and facilities for arterial repair and grafting must be at hand to minimize the chances of stroke.^6)^ We also used intraluminal shunt in 13.6% (n=3) of cases to prevent stroke in our patients. Transient neuropraxia of the hypoglossal nerve resolving within 3 months was noted in 13.6% (n=3) of cases, while permanent drooping of the lower lip was noted in 4.5% (n=1). None of our patients had stroke peroperatively or postoperatively. Malignant potential in CBTs is 5%–7%.^3,10)^ In our study, none of the patients had clinical evidence of local/distant metastasis, and histopathology reports confirmed about their benign nature.

Gilbo et al., in their study of 156 cases, advocated that patients who are unfit for surgery or refuse surgery should be treated with radiotherapy as an alternative treatment option.^20)^ In our series, we reserved this modality for a recurrent tumor.

The retrospective nature and small number of cases in our study are potential limitations. However, the rarity of this tumor should be kept in mind while assessing these limitations.

## Conclusion

Complete surgical excision of primary CBTs at an early stage, regardless of its size and Shamblin class, is associated with the best outcome. Chances of concomitant nerve injury peroperatively are higher in Shamblin class III tumors.
